# Prolongation of length of stay and *Clostridium difficile* infection: a review of the methods used to examine length of stay due to healthcare associated infections

**DOI:** 10.1186/2047-2994-1-14

**Published:** 2012-04-20

**Authors:** Brett G Mitchell, Anne Gardner

**Affiliations:** 1 School of Nursing, Midwifery and Paramedicine, Australian Catholic University, PO BOX 256, Dickson, ACT, Australia; 2 Research Associate, National Centre for Clinical Outcomes Research (NaCCOR), Australian Catholic University, Sydney, Australia

**Keywords:** *Clostridium difficile* infection, *Clostridium difficile* associated diarrhoea, Cost, Healthcare associated infection, Length of stay, Time dependent bias

## Abstract

**Background:**

It is believed that *Clostridium difficile* infection (CDI) contributes to a prolongation of length of stay (LOS). Recent literature suggests that models previously used to determine LOS due to infection have overestimated LOS, compared to newer statistical models. The purpose of this review is to understand the impact that CDI has on LOS and in doing so, describe the methodological approaches used.

**Aim:**

First, to investigate and describe the reported prolongation of LOS in hospitalised patients with CDI. Second, to describe the methodologies used for determining excess LOS.

**Methods:**

An integrative review method was used. Papers were reviewed and analysed individually and themes were combined using integrative methods.

**Results:**

Findings from all studies suggested that CDI contributes to a longer LOS in hospital. In studies that compared persons with and without CDI, the difference in the LOS between the two groups ranged from 2.8days to 16.1days. Potential limitations with data analysis were identified, given that no study fully addressed the issue of a time-dependent bias when examining the LOS. Recent literature suggests that a multi-state model should be used to manage the issue of time-dependent bias.

**Conclusion:**

Studies examining LOS attributed to CDI varied considerably in design and data collected. Future studies examining LOS related to CDI and other healthcare associated infections should consider capturing the timing of infection in order to be able to employ a multi-state model for data analysis.

## Background

*Clostridium difficile* infection (CDI) is the leading cause of infectious diarrhoea in hospitalised patients [1]. According to the Centers for Disease Control and Prevention (CDC), the annual incidence of CDI in the USA exceeds 250 000 hospitalised cases, with a mortality of 12.5% [2]. The diseases symptoms can range from colonisation to life-threatening colitis. The incidence of morbidity related to CDI is increasing due to an epidemic of a hypervirulent strain of *C.difficile* (BI/NAP1) that has been reported in the USA and other countries. In addition to significant morbidity and mortality, CDI increases healthcare costs due to patients extended hospitalisations and re-hospitalisations [3]. A recent systematic review investigating the economic costs to healthcare associated with CDI concluded that despite a lack of common methods employed by the studies, it is clear that the economic consequences of CDI are considerable [4].

One important step towards understanding the burden that CDI has on the health service is to examine the economic cost of CDI in hospitalised patients. One of the major costs associated with any healthcare associated infection (HAI) is excess hospitalisation, or prolongation of length of stay (LOS). A challenge for researchers is to design a study that accurately accounts for prolonged lengths of stay. Recent literature suggests that models that have been previously used to determine the additional LOS in hospital due to infection overestimated the additional LOS, compared to newer statistical models [5-10]. It is therefore vital that studies are designed in such a way as to evaluate and analyse this effectively. Determining the additional LOS due to an HAI, including CDI, is challenging due to the need to manage time-dependent biasthat is, the longer a person stays in hospital, the greater the risk of acquiring an infection. Time dependent bias is a term used to describe problem occurring when variables in the model change value after the start of patient observation. Such variables are called time dependent, because their value can change over time [11]. One study demonstrating this bias examined readmission hospital and whether persons with a discharge summary were followed up by a physician after discharge. When the time dependent variable was analysed as a fixed variable, there were significantly lower readmissions in patients who saw physicians with the summary. This was shown to be a biased association as patients with early hospital readmission did not have a chance to see a physician and these patients were placed in a non discharge summary group [12]. There are numerous other publications which also demonstrate this issue [7-9,13,14]

Therefore, managing issues such as time-dependent bias and sampling bias are important. The purpose of this review is to understand the impact that CDI has on LOS in hospitalised patients and, in doing so, to describe the methodological approaches used.

## Methods

### Design

An integrative review design was used in the same manner as described by Whittemore and Knafl [15]. To allow for a synthesis of results, an integrative design was selected based on the summation of different methodological approaches used in the empirical and theoretical literature. As a result, the design provides a more comprehensive understanding of particular issues [15].

### Search strategy

The literature was accessed through searches on electronic databases *Medline* and *Pubmed* and was limited to the 1^st^ January 2000 to 30 April 2011. Other limits included only searching literature that was published in English and studies involving humans. Key terms used were *Clostridium difficile* and economic, *Clostridium difficile* and length of stay, *Clostridium difficile* and cost and *Clostridium difficile* and burden. These searches were combined, with duplicate studies being removed. Following this step, a review of these articles was conducted. Only case-controlled, cohort or review studies were included. Furthermore, articles were only included if they examined the LOS of hospitalised patients with CDI. Finally, letters to the editor and interventional studies, for example the effect of immunoglobulin treatment on LOS, were excluded.

### Search outcome

**Figure 1:**
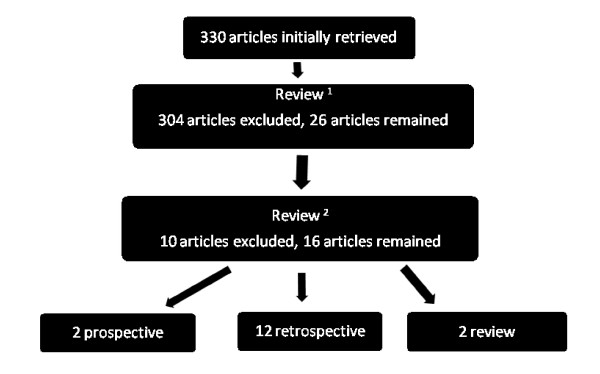
**Summary of search strategy.**^1^Articles were excluded if they were not case controlled, cohort or review studies or if they did not examine length of stay in hospitalised patients. ^2^Letters to the editor and interventional studies were excluded.

The initial search yielded 330 articles. After the removal of studies that were not case-controlled, cohort or review studies, 26 studies remained. A further ten articles were excluded because they were either letters to the editor or were interventional studies. Figure 1 summarises the search strategy and outcomes.

## Results

The majority of the 16 studies identified through the search strategy were retrospective in design. Two reviews and two prospective studies were identified. Table 1 summarises the characteristics and results from the 16 studies identified for this review.

**Table 1:** Summary of included articles

Author	Study type	Country	Statistical analysis	Results
Ananthakrishnan, McGinley, & Binion 2008	Retrospective case control	US	Multivariate regression	Three times the length of stay (CDI+IBD) vs. controls (IBD) IBD=irritable bowel disease
Bajaj et al. 2010	Retrospective case control	US	Multivariate regression	12.7-day case vs. 6.7-day control
Dubberke, Butler et al. 2008	Retrospective cohort	US	Multivariate regression, matched-pairs analysis	9.6-day cases vs. 5.8-day controls
Dubberke & Wertheimer 2009	Review	Not applicable	Not applicable	Not applicable
Ghantoji et al. 2010	Review	Not applicable	Not applicable	Not applicable
Kenneally et al. 2007	Retrospective cohort	US	Multiple logistic regression	27.3 (CDI) vs. 22.8 (non CDI)
Lawrence et al. 2007	Retrospective cohort	US	Multiple logistic regression	CDI stay twice as much non-CDI in ICU
Lumpkins et al. 2008	Prospective cohort	US	Multiple logistic regression	34.9day LOS with CDI vs. 19 LOS without CDI
Miller et al. 2002	Retrospective cohort	Canada	Not discussed	9% of 269 patients with CDI deemed to have extension of LOS due to CDI
Nguyen, Kaplan, Harris, & Brant 2008	Retrospective cohort	US	Multiple linear regression	65% increase in LOS in patients with CDI & Crohns disease 46% increase in LOS in patients with CDI & ulcerative colitis
OBrien, Lahue, Caro, & Davidson 2007	Retrospective cohort	US	Descriptive	6.4-day LOS in patients with primary CDI diagnosis
Pepin, Valiquette, & Cossette 2005	Retrospective case control	Canada	Not discussed	10.7-day additional LOS in patient with CDI
Song et al. 2008	Retrospective match cohort	US	Logistic regression Wilcoxon Linear regression	1-day LOS increase (with CDI)
4-day LOS increase (CDI) when compared to matched diagnosis related group DRG				
Vonberg et al. 2008	Prospective match cohort	Germany	Wilcoxon Kolmogorov-Smirnov test	27-day LOS cases vs. 20-day LOS controls
Zerey et al. 2007	Retrospective cohort	US	Multiple logistic regression	16-day-longer LOS (with CDI)
Zilberberg et al. 2009	Retrospective cohort	US	Propensity score Multivariate analysis	6.1-day longer LOS (with CDI)

The search strategy used to identify articles for this review did not identify the same articles in the latest review published by Ghantoji, Sail et al. [4]. Two articles included in the review by Ghantoji, Sail et al. [4] were not include in our review. Conversely, our study identified and included eight studies not used by Ghantoji, Sail et al. [4]. The primary reason for both these discrepancies is that our review examined the prolongation of LOS, whereas the focus by Ghantoji, Sail et al. [4] was economic cost . Similarly our review did not include two articles identified by the review conducted by Dubberke & Wertheimer (2009), but did identify a further 11 articles not used by Dubberke & Wertheimer (2009). The reasons for this are the same as those just previously described in addition to the inclusion of recent publications. Nine articles were common to both reviews. The review by Ghantoji, Sail et al. [4] identified four articles not identified by Dubberke & Wertheimer (2009). Conversely, Dubberke & Wertheimer (2009) identified five articles not used by Ghantoji, Sail et al. [4].

The manner in which participants were identified for the studies differed, with several studies using International Classification of Disease (ICD) codes to identify cases [16-21]. The use of ICD codes to identify participants does have the potential to reduce sensitivity and specificity when identifying cases of CDI as coding data is likely to underestimate cases. In addition, coding practices can vary between hospitals, and therefore multi-centred studies have a greater potential for variation in sample selection. Furthermore, the timing of an episode of CDI cannot be determined by such an approach.

Excluding the reviews, only three of the remaining fourteen studies were undertaken in countries other than the United States. The systematic review examining the economic costs of CDI undertaken by Ghantoji, Sail et al. [4] identified only four of thirteen articles from the United States. In the review undertaken by Dubberke and Wertheimer [22], one Australian study undertaken was identified as having been published as a letter to the editor [23].

The data collected in the various studies differed considerably. The majority of studies collected basic demographic data, such as age and gender. Some studies collected data about co morbidities and used a severity index such as the Charlson co morbidity index [18,24,25]. Data collected about variables such as antibiotic exposure or other drug therapies were limited [25-27].

Findings from all studies suggested that CDI contributes to a longer LOS in hospital. It was not possible to pool data because studies varied considerably in design, sampling and data analysis techniques. In studies that used a comparison between persons with CDI and those without, the difference in the LOS between the two groups ranged from 2.8days to 16.1days [24,28]. These data suggest that CDI does play a role in increasing the LOS in hospital.

In a retrospective cohort of over 18 000 non-surgical patients hospitalised for more than 48 h, Dubberke et al. [24] took a nested subset using a matched-pairs analysis and found that the increase in LOS that could be attributed to CDI was 2.8days. Controls were matched to cases by a propensity score developed for data analysis. Using logistic regression, patient-specific probabilities of developing CDI were developed. The median LOS was determined for cases and controls, with the various median pair-wise lengths of stay being compared by using the Wilcoxon signed-ranked test. Attributable LOS was determined by calculating the median pair-wise difference between CDI cases and the controls [24]. As this study did not include surgical patients, it is possible that patients with severe CDI, those requiring colectomies, were excluded, leading to a potential bias. The use of a propensity score to match controls was used in an attempt to reduce any potential bias between controls and cases when determining CDI-attributable LOS.

A study undertaken by Lumpkins et al. [28] suggested a considerably longer LOS then that reported by Dubberke et al. [24]. In a prospective cohort study comprising of critically ill patients admitted to an intensive care unit, those with and without CDI were compared. A logistic regression model was used for data analysis. The mean hospital LOS was 15.9days greater in patients who developed CDI compared to those who did not (34.9days versus 19.0days, p=0.003). When cases were compared regarding antibiotic exposure, those with minimal exposure were found to have a shorter LOS in hospital, but data regarding all antibiotic exposure prior to admission, such as outpatients, were not obtained in this study [28]. Such a finding would suggest that collecting data on antibiotic exposure is needed in future studies that employ a similar methodology.

The methods of data analysis varied, as shown in Table 1. In the majority of studies, a regression model was developed to determine the impact that CDI had on LOS [16,18,20,21,24,26-30]. The studies did not collect data concerning the time of onset of CDI; therefore, it is not possible to exclude the possibility of reverse causality, in which longer lengths of hospitalisation may have increased the risk of CDI. The issues associated with controlling for a potential time-dependent bias caused by the LOS in hospital raises some significant concerns, which will now be discussed.

## Discussion

As demonstrated in a published systematic review examining the economic costs of CDI, the focus of many studies was to view costs through the eyes of an accountant [4]. An accountants model for determining the cost of HAIs is to count fixed and variables costs. Variable costs may include items such as dressings, personal protective equipment and laboratory test materials. Fixed costs include salary, electricity and heating. As fixed costs are often jointly sharedfor example, one doctor does not treat one patientthe accountants model determines a measure of usage for these fixed costs (cost per unit) and allocates this to patients or to the health provider accordingly. Comparisons between the average cost per infected patient and average cost per non-infected patient are often used to attribute the cost of HAIs. However, this may be misleading [31]. According to Graves, using such a model is not suitable for economic appraisal or for informing decisions about HAIs. An implication of the economic model is that by reducing or eradicating a specific infection, a fixed figure could be saved. An accountants model ignores the cost of increased investments towards reducing infections and fails to consider which costs actually change with infections, as many fixed costs remain [31].

An economist model uses a cost-analysis approach to determine if there are any savings. For example, the consumables may be reduced by decreasing the instances of HAIs. The capacity gained by a reduction in HAIs is valuable and should thus be redeployed for other use. The redeployment of resources could be used for tasks such as elective surgery and, in turn, could cause other variable costs to increase [31].

An economists approach in evaluating the cost of HAIs is supported by the argument that the majority of the costs associated with hospital care are fixed [32,33]. Therefore, in describing how costs change in relation to HAIs, it is important to demonstrate the number of bed days caused by HAIs [31]and therefore the number of beds that are made available by preventing these infectionsbefore deciding who will utilise these extra beds. Accurately determining the prolongation of LOS due to CDI will assist in developing an economic model for its prevention and control.

All of the studies identified in this literature review suggested that CDI contributes to a longer LOS in hospital. However, the method used to determine LOS should account for the fact that an HAI, such as CDI, can occur at any point during hospitalisation and that LOS is affected by other variables, such as co morbidity and primary diagnosis [5]. Matched cohort studies suffer from two types of bias. First, insufficient matching will not control all the bias. Second, strict matching criteria will result in censoring. The variable nature of when the infection might have started also poses an issue in matched studies: infections can occur at any time. However, data analysis in matched studies often compared infected and uninfected patients by their total hospital stay. If the timing of infections is not taken into account, then costs associated with pre- and post-infection are included and can dramatically amplify the time-dependent bias [5]. Statistical models can be used to address this issue at the data-analysis stage rather than at the design stage. A model can be built to describe the relationship between LOS and the predictors of that outcome [5,34]. Previously, models that ignored the time of infection often used a linear model that assumed a gamma distribution, where waiting times between events are relevant, in this case LOS and an independent variable of infected (yes or no) [14]. One recent study examining CDI did attempt to use the principles of managing time-dependent bias in their study [35].

Methods have recently been developed to address these issues when estimating LOS associated with healthcare-associated infections. These methods include a multi-state model in which the infection is the intermediate event between admission and discharge and in which patients are given one of three states: non-infected, infected and discharged [6,14,36]. Therefore, for future research examining the prolongation of LOS for people with an HAI including CDI, collecting data at the commencement and completion of infection will enable the use of a multi-state model in data analysis.

## Conclusion

Studies examining lengths of stay attributed to CDI varied considerably in their design and the data they collected. Several studies used administrative codes, such as ICD codes, to identify cases of CDI. The use of administrative data for this purpose did lead to some limitations, including the potential for ascertainment bias and a lack of sensitivity and specificity. A limited number of studies captured data regarding co morbidities. Co morbidities would clearly influence the LOS in hospital, and therefore this information should be collected when possible. Researchers, should consider whether data concerning antibiotic exposure needs to be included in future studies.

Despite these differences, there was a clear indication that CDI played some role in prolonging hospitalised patients lengths of stay. As LOS in a hospital is a major contributor to healthcare cost, it is a logical assumption that CDI contributes an economic cost to the health system, a view shared by Ghantoji, Sail et al. [4]. Only a very limited number of studies identified in this literature review or in the two published reviews by Ghantoji, Sail et al. [4] and Dubberke and Wertheimer [22] did so outside of the United States or Canada. The provision of health services and the epidemiology of CDI varies between countries, and thus it is vital that future studies are undertaken in a variety of countries. In particular, studies outside of the United States and Canada are needed.

Potential issues in data analysis were identified, given that no study fully addressed the issue of a time-dependent bias when examining the LOS caused by CDI. Recent literature suggests that a multi-state model should be used to manage the issue of time-dependent bias. In order for a multi-state model to be used, the timing of CDI infection must be captured. However, no study identified in the literature search, including the two published reviews examining the economic cost of CDI, used or identified a multi-state model design. In fact, no study identified the onset and cessation of CDI infection and used this data to inform data analysis. Future studies examining LOS and CDI should consider capturing the timing of CDI infection in order to be able to employ a multi-state model for data analysis. Such an approach can also be extended in order to study HAIs other than CDI.

## Competing interests

The authors declare they have no competing interests.

## Author contributions

BM and AG were responsible for the study concept and design. BM performed data collection. BM and AG were responsible for data analysis and the draft of the manuscript. All authors have read and approved the manuscript.
